# Brain Linac-Based Radiation Therapy: “Test Drive” of New Immobilization Solution and Surface Guided Radiation Therapy

**DOI:** 10.3390/jpm11121351

**Published:** 2021-12-12

**Authors:** Fabiana Gregucci, Ilaria Bonaparte, Alessia Surgo, Morena Caliandro, Roberta Carbonara, Maria Paola Ciliberti, Alberto Aga, Francesco Berloco, Marina De Masi, Christian De Pascali, Federica Fragnoli, Chiara Indellicati, Rosalinda Parabita, Giuseppe Sanfrancesco, Luciana Branà, Annarita Ciocia, Domenico Curci, Pietro Guida, Alba Fiorentino

**Affiliations:** Department of Radiation Oncology, Miulli General Regional Hospital, 70021 Acquaviva delle Fonti-Bari, Italy; fabianagregucci@gmail.com (F.G.); ilariabonaparte@libero.it (I.B.); morenacaliandro@yahoo.it (M.C.); roberta.carbonara@yahoo.it (R.C.); mpciliberti@gmail.com (M.P.C.); albertoaga918@gmail.com (A.A.); francesco.berloco13@gmail.com (F.B.); marinademasi97@gmail.com (M.D.M.); ch.depascali@libero.it (C.D.P.); federica.fragnoli@gmail.com (F.F.); indellicatichiara@gmail.com (C.I.); rosalinda.parabita@gmail.com (R.P.); g.sanfrancesco@gmail.com (G.S.); branaluciana@gmail.com (L.B.); annaritaciocia@gmail.com (A.C.); domenico.curci7@gmail.com (D.C.); p.guida@miulli.it (P.G.); albafiorentino@hotmail.it (A.F.)

**Keywords:** surface guided radiation therapy, linear accelerator-based radiotherapy, brain, radiosurgery

## Abstract

Aim: To test inter-fraction reproducibility, intrafraction stability, technician aspects, and patient/physician’s comfort of a dedicated immobilization solution for Brain Linac-based radiation therapy (RT). Methods: A pitch-enabled head positioner with an open-face mask were used and, to evaluate inter- and intrafraction variations, 1–3 Cone-Beam Computed Tomography (CBCT) were performed. Surface Guided Radiation Therapy (SGRT) was used to evaluate intrafraction variations at 3 time points: initial (i), final (f), and monitoring (m) (before, end, and during RT). Data regarding technician mask aspect were collected. Results: Between October 2019 and April 2020, 69 patients with brain disease were treated: 45 received stereotactic RT and 24 conventional RT; 556 treatment sessions and 863 CBCT’s were performed. Inter-fraction CBCT mean values were longitudinally 0.9 mm, laterally 0.8 mm, vertically 1.1 mm, roll 0.58°, pitch 0.59°, yaw 0.67°. Intrafraction CBCT mean values were longitudinally 0.3 mm, laterally 0.3 mm, vertically 0.4 mm, roll 0.22°, pitch 0.33°, yaw 0.24°. SGRT intrafraction mean values were: i_, m_, f_ longitudinally 0.09 mm, 0.45 mm, 0.31 mm; i_, m_, f_ laterally 0.07 mm, 0.36 mm, 0.20 mm; i_, m_, f_ vertically 0.06 mm, 0.31 mm, 0.22 mm; i_, m_, f_ roll 0.025°, 0.208°, 0.118°; i_, m_, f_ pitch 0.036°, 0.307°, 0.194°; i_, m_, f_ yaw 0.039°, 0.274°, 0.189°. Conclusions: This immobilization solution is reproducible and stable. Combining CBCT and SGRT data confirm that 1 mm CTV-PTV margin for Linac-based SRT was adequate. Using open-face mask and SGRT, for conventional RT, radiological imaging could be omitted.

## 1. Introduction

Radiation therapy (RT) plays a key role in the treatment of several Central Nervous System (CNS) oncological and non-oncological diseases, with growing attention for stereotactic radiotherapy (SRT) [1]. Historically, the path of primary brain cancer cure, of which glioblastoma (GBM) is the most common type, requires RT according to Stupp’s trial and, recently, reirradiation finds a place in cases of disease recurrence [2,3]. In the same way, SRT is an evolving paradigmatic approach for the treatment of brain metastases (BMs) [4]. Moreover, radiosurgery is also increasingly used in the treatment of benign lesions such as meningiomas and vascular malformations [5].

Non-invasiveness, precision, safety and efficacy are the elements that over the years have allowed RT to achieve these therapeutic goals. With the continuous improvement of techniques and technologies available today, the purpose of modern RT is to reduce the exposure of healthy tissues to irradiation and improve the accuracy in delivering of therapeutic doses to target lesion. A crucial point for Linac-based RT to reach this progress is the optimal definition of *“*safety margins” from Gross Tumor Volume (GTV) or Clinical Target Volume (CTV) to Planning Target Volume (PTV), whose amplitude considers the setup errors [6]. Inevitably, to reduce planning margins, first, this process must go through the improvement of the immobilization systems to ensure the correct and reproducible positioning of the patient at the beginning and during RT and, at the same time, the possibility of monitoring the correct maintenance of this position during RT delivery. Second, evaluating immobilization systems, two further aspects should be considered: (i) comfort for patient and (ii) ease and time of device realization by health practitioners, due to the fact that these factors could influence product reliability and patient’s treatment compliance.

For the treatment of intracranial lesions several dedicated invasive and noninvasive immobilization systems were compared to evaluate patient intra- and inter-fraction head motion [7]. Furthermore, the possibility of using a thermoplastic open-face mask appeals to the idea of using a surface imaging system as a form of noninvasive and nonradiographic image guidance to accurately monitor and quantify movement throughout the entirety of treatment live [8].

Based on this background, primary aim of this prospective exploratory observational study was to test inter-fraction reproducibility, intrafraction stability, technician aspects and patient/physician’s comfort of new dedicated immobilization solution for treatment of intracranial disease with Linac-based RT. Moreover, in cases of SRT treatments, data regarding real-time Surface Guided RT (SGRT) were collected with the secondary aim to evaluate intrafraction accuracy.

## 2. Materials and Methods

In the present prospective observational study, the data regarding inter- and intrafraction variations of the Solstice^TM^ SRS Immobilization System, CIVCO^®^ Radiotherapy (Orange City, FL, USA) using imaging pre- and post-RT session were collected, to evaluate reproducibility and stability for Linac-based precise and modern RT.

Data regarding technician aspects (such as ease of use and stability of pitch locking lever/mask clips, therm-shrinkage, total sim setup time) and patient/physician’s comfort were also considered. In cases of SRT treatments, data regarding SGRT were collected using Optical Surface Monitor System AlignRT^®^.

The inclusion criteria were: (i) age > 18 years; (ii) diagnosis of CNS disease suitable of RT; (iii) informed consent.

### 2.1. Immobilization Device and Simulation Computed Tomography (Sim-CT)

The Solstice system, comprised of a carbon fiber head support, customizable accuform cushion and thermoplastic open-face mask. The head support allows manual pitch setup errors correction by rotating the screw located at the back of the system. Three distinct landmarks were positioned to the mask (1 frontal and 2 laterals). Sim-CT was performed without contrast and the scan length included the whole brain, acquiring slices of 1–3 mm thickness, depending on different planned treatment.

Two radiation-therapists (RTTs) were responsible for the construction of the thermoplastic mask and customizable cushion for each patient during sim-CT procedures. At the end of each procedure RTTs recorded technician aspects in a specific form scored in 4 levels (poor/fair/good/excellent) relating to: (1) pitch locking level (ease of use, ease of locking indentation and stability of lock), (2) mask clips (ease of use).

The total simulation setup time (SST) was calculated, including all the required procedures: from the recline patient position on the sim-CT couch to preparation of the cushion, molding and cooling down of the mask, and finally the acquisition of CT images.

After sim-CT, a radiation oncologist interviewed the patient to collect information about comfort, focusing on therm-shrinkage. Moreover, physician’s feedback experience was recorded, equally, scored in poor, fair, good or excellent.

### 2.2. Target Volume Definition and Treatment Planning

The target volume definition was different according to diagnosis and planned radiation treatment. In primary brain cancer, the GTV was defined as published in international guidelines [9]. For whole brain radiotherapy (WBRT) in cases of multiple BMs, CTV was the entire brain, adding a 5 mm isotropic margin to define the PTV. For SRT, in cases of limited BMs or primary brain cancer recurrence or benignant brain lesions, GTV coincided with CTV and was defined based on T1 contrast sequence of MRI. The PTV was defined adding an isotropic margin of 1 mm, excluding lesions into brainstem in which no margin was given from the CTV to the PTV, according to international guidelines [10,11]. The dose prescriptions were different according to the therapeutic approach: conventional RT (cRT) versus SRT. For primary brain cancer the prescription dose was chosen between 60 or 40 Gy in 30 or 15 fractions, respectively [2,12] according to treatment volumes and patient’s performance status. For WBRT the prescription dose was 20 or 30 Gy (4 or 3 Gy per fraction). For SRT, the dose prescription ranged between 12–24 Gy in 1 fraction or 21–27 Gy in 3 fractions or 25–30 Gy in 5 fractions, according to histology, PTV volume, proximity to the organs at risk and patient’s clinical evaluation [3,5,13]. Flattening Filter Free (FFF) beam and Volumetric Modulated Arc Therapy (VMAT) with 2 or more coplanar or non-coplanar arcs, optimized for a TrueBeam™ (Varian Medical System), were generated for each plan. Dose prescription, normalization and optimization were according to ICRU83 and ICRU91 guidelines.

### 2.3. CBCT-SGRT Workflow and Data Collections

The patients were placed in each treatment session using SGRT and, subsequently, the current position was checked using CBCT.

To test inter-fraction reproducibility and intrafraction stability, the online imaging procedures using Cone-Beam CT (CBCT) were different between cRT and SRT.

In cases of cRT, a single CBCT was acquired before each RT session. A rigid match between sim-CT- and CBCT images was performed automatically by a software, using cranial bones as focus point and validated by an expert radiation oncologist and RTTs.

In cases of SRT, 3 CBCT were acquired: one before treatment on which a first rigid match setup was performed and shifts applied, a second one (II-CBCT) to confirm shifts and third (III-CBCT) to verify position at the end of SRT to identify any patient movements during delivery (intrafraction motions).

The setup error tolerance was different in cRT or SRT treatments. In the conventional fractionation shift tolerance in translational and rotational inter-fraction motions were ≤5 mm and 2°, while ≤1 mm and 1° was applied in SRT treatments. In all cases a 6D correction was executed. If the tolerance was exceeded, the patient was repositioned, and the entire procedure was repeated.

Regarding SGRT workflow, the open-face portion of the mask was wired during simulation to enable viewing of the open-face region within the treatment planning system. The open-face region was then contoured, the eye regions removed, and the resulting structure was exported to AlignRT^®^ to be used as the region of interest (ROI) for SGRT. Before treatment, the RTTs used SGRT to adjust patient positioning. After this procedure, in all cases, first CBCT was used to verify correct patient position. After CBCT alignment, a reference surface was captured in AlignRT^®^ and treatment was initiated. During treatment, the patient’s movements were monitored with surface images (SI) offsets, called Real-Time Deltas (RTDs) in AlignRT^®^. If the magnitude (MAG) of translational RTDs exceeded patient-specific thresholds (typically 1 mm), the delivery was automatically stopped, resuming when MAG was in range. The CBCT and SGRT data collections were performed off-line for each treatment session.

Match values of all three translational axes (x = lateral, y = longitudinal, z = vertical) and three rotational axes (roll, pitch and yaw) were recorded. The inter-fraction variability was obtained by matching the first CBCT with planning-CT. The intrafraction variability was obtained by matching the post-treatment CBCT (III-CBCT) to the CBCT acquired right before treatment delivery (II-CBCT). Moreover, for each treatment session, SGRT was applied to evaluate intrafraction variations, acquiring data in 3 different time points: initial (i), final (f), and monitoring (m) (before, at the end, and during treatment, respectively).

### 2.4. Statistical Analysis

The study was designed as a prospective exploratory observational trial to evaluate the standard deviation and relative confidence interval of 95% (95% CI) for each variable linked to test reproducibility and stability of the immobilization system under question. In accordance with this aim, a statistical analysis was performed regarding inter- and intrafraction variability based on CBCT data and intrafraction variability based on SGRT data, respectively for each translational and rotational values. Collected data were considered to be quantitative continuous variables and their distributions were evaluated with the appropriate indexes of centrality and variability: mean, standard deviation (SD) with relative confidence interval of 95% (95% CI) and quartiles (25%, 50% and 75%). For each variation, the absolute value was calculated as deviation from zero. Data management and statistical analysis were conducted using Stata Statistical Software (Release 16. College Station, TX, USA: StataCorp LLC).

## 3. Results

### 3.1. Cohort of Study

Between October 2019 and April 2020, 69 patients (21 female and 48 male) were treated for a total of 556 treatment session and 863 CBCT. The median age was 66 years (range 27–89 years). Between sim-CT and treatment, a median of 8 days (range 2–15 days) occurred. Regarding diagnosis: BMs and primary brain cancer were the most frequent (Table 1).

Twenty-four (35%) cases received cRT with a median dose of 40 Gy (range 20–60 Gy) [14]. SRT were performed in 45 cases (65%) with a median dose of 27 Gy (range 12–30 Gy), median fraction of 3 (range 1–5) and median target volume of 6.15 cc (range 0.7–104 cc). The main characteristics of study population were summarized in Table 1.

### 3.2. Inter-Fraction Reproducibility and Intrafraction Stability

**Figure 1:**
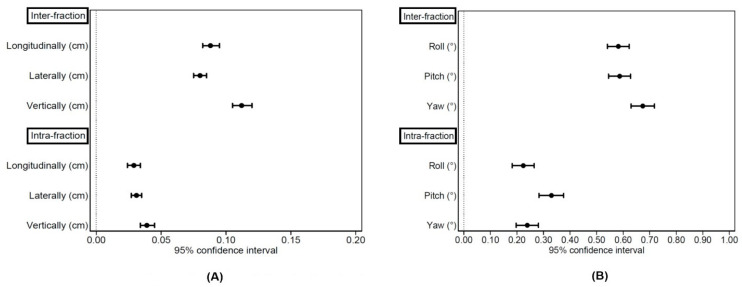
95% CI for inter- and intrafraction (**A**) translational and (**B**) rotational values.

Inter-fraction CBCT mean values (±SD), for whole study population in all translational and rotational directions were longitudinally 0.9 mm (±0.8 mm), laterally 0.8 mm (±0.6 mm), vertically 1.1 mm (±0.9 mm), roll 0.58° (±0.49°), pitch 0.59° (±0.49°), yaw 0.67° (±0.53°). The main summary statistical indices were reported in Table 2. Intrafraction CBCT mean values (±SD) for SRT study group in all translational and rotational directions were longitudinally 0.3 mm (±0.3 mm), laterally 0.3 mm (±0.3 mm), vertically 0.4 mm (±0.3 mm), roll 0.22° (±0.26°), pitch 0.33° (0.29°), yaw 0.24° (±0.26°). The main summary statistical indices were reported in Table 3. In Figure 1 the 95% CI were showed for inter- and intrafraction values.

**Figure 2:**
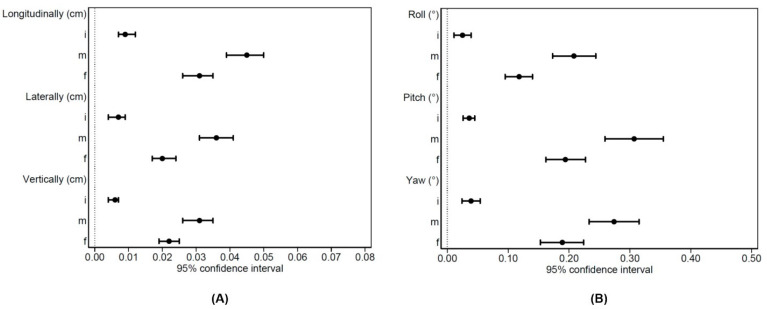
95%CI for SGRT (**A**) translational and (**B**) rotational values.

Data regarding SGRT were reported in Table 4. In Figure 2 the 95% CI were showed for SGRT values.

### 3.3. Technician Aspects, Patient’s and Physician’s Evaluations

In all cases, RTTs reported good and excellent score relating to whole technician aspects: (1) pitch locking level (ease of use, ease of locking indentation and stability of lock), (2) mask clips (ease of use). The median SST was 12 min (range 10–18 min). Similarly, patient’s and physician’s comfort were assessed with good and excellent score in all cases.

## 4. Discussion

Historically, brain radiotherapy focusing on stereotactic treatment was introduced by Larsel Leksell that in collaboration with Borge Larson created the first Gamma Knife^®^ (GK). GK represents today the gold standard to perform SRT, allowing a highly performing precision of the treatment, partly due to the rigid and invasive immobilization system used [15]. However, those that from a certain point of view are advantages, could also be considered the major disadvantages of GK-based-SRT. Due to the technological advancement, including frameless and patient’s comfort, interest in Linac-based-SRT is increasing considerably [3,4,5,6,10,11,12,13,14,15]. In this context, the current identification of margins from CTV to PTV that accounts for organ motion and deformation and for setup uncertainties due to patient daily positioning variations, is crucial. If target coverage is not compromised, volume de-escalation may translate into a reduction of side effects, improvement of quality of life and improved of cost-effectiveness in healthcare [6]. In line with these assumptions, the present analysis was designed to test reproducibility and stability of new dedicated noninvasive stereotactic immobilization device (Solstice^TM^ SRS Immobilization System, CIVCO^®^ Radiotherapy) and to identify PTV-safety margins and adequate workflow for Linac-based-SRT, combining CBCT and SGRT data.

At first, inter-fraction CBCT mean values (±SD) were analyzed in all translational and rotational directions and relative IC95% found that motions were <1 mm and <1°, respectively. Therefore, considering the position procedure consisting of the patient placement in each treatment session using SGRT and, subsequently, its check using CBCT, it is possible to deduce that SGRT is useful and safe to address the patient into a submillimeter position also without taking CBCT. Subsequently, the same evaluations were conducted on intrafraction CBCT highlighting these translational values were <0.05 mm and rotational values <0.5°. These results were consistent with the study published by Ong CL et al. [16] that evaluated the setup accuracy of the same immobilization device in a small cohort of patients treated in two different institutes. The authors [16] analyzed 33 patients for a total of 63 pre- and 63 post-CBCT showing that for translational and rotational directions, the mean inter-fraction motion was inferior to 1mm and 0.5°, respectively, while the mean intrafraction motions for all translational and rotational direction were <0.2 mm and 0.5°; however, no confidence intervals were provided for the sample estimate.

The results of the present analysis performed on 69 patients for a total of 556 inter-fraction and 307 intrafraction CBCT confirmed Ong CL data. The analysis of 95% CI showed the robustness and reliability of the data. In fact, in accordance with the study aim, the present trial was not designed as a power study and the sample estimate was not required. However, considering the large sample size, the SD value for explorative variables and the relatively small width of IC95%, the estimates obtained can be considered statistically significant.

Based on these evaluations, Solstice^TM^ SRS Immobilization System ensures that setup errors and possible movements that the patient may perform during treatment are minimized and included in the CTV-PTV margin of 1 mm. Several studies aimed to evaluate different noninvasive immobilization and relative CTV-PTV margin for Linac-based-SRT [7,16,17,18,19,20] showed a safety margin ranged between 1–3 mm. Overall, international guidelines [11,20,21,22] support the use of noninvasive immobilization techniques to perform with high accuracy SRT. However, the same guidelines [21,22] do not provide definitive indications on the optimal workflow to monitor the treatment during delivery, although the use of image guidance including kV orthogonal pair and CBCT is recommended. In our study, for SRT treatment session, a workflow with 3 CBCT was applied considering the narrow margin used and the movements of the couch. In fact, to perform a high-conformed treatment field, multiple non-coplanar VMAT arches with different angles of rotation of the coach couch (median 4) were used, requiring a median overall treatment time of 2 min. CBCT data showed that intrafraction shifts were submillimeter. Moreover, to improve the accuracy of setup and to follow the patient during delivery, thanks to the use of open-face mask, it was possible to apply an SGRT protocol. The analysis of intrafraction SGRT data agreed with pre- and post-treatment CBCT findings. These results suggest a workflow for assessing the accuracy of the treatment in the delivery phase that may include a pre-treatment CBCT to correct setup errors and subsequent monitoring and final control with SGRT, omitting the CBCT to confirm and to verify the correct target position at the end of the treatment session. Considering the analysis of inter-fraction CBCT and SGRT data, for conventional treatment in which a larger CTV-PTV margin (5 mm) is used than SRT, it may be reasonable to use only SGRT in conjunction with the Solstice^TM^ SRS Immobilization System open-face mask for patient positioning, verification and monitoring, reducing the number of radiological imaging (kV orthogonal pair or CBCT) from daily to weekly. This approach would reduce the overall treatment time by increasing the comfort for the patient, without detriment to the quality of the therapy. In this way, during recent years, a growing literature supports the use of optical surface guidance for patient positioning and monitoring in several clinical situations, including intracranial SRT [23,24].

Finally, we took into consideration patient’s and TTs’ comfort with this new immobilization device, evaluating that patient- and user-friendly mask could represent an important advantage to overcome the limitations of frame-based SRT and to improve planning and treatment compliance. Our results showed good and excellent score relating to confidence and satisfaction aspects.

## 5. Conclusions

The present analysis showed that this new immobilization solution allows use of very slight 1 mm CTV-PTV margin for Linac-based SRT and ensures reproducibility and stability of the system for the entire treatment duration, reporting excellent characteristics in terms of ease of procedures and patient/physician’s comfort. Combining CBCT and SGRT data, results confirm the accuracy of Linac-based RT. Using open-face mask and SGRT, for cRT, radiological imaging (portal or CBCT) could be omitted.

## Figures and Tables

**Table 1:** Main characteristics of study population.

Total Patients	69
cRT group	24 (35%)
SRT group	45 (65%)
Total treatment session	556
cRT treatment session	397 (71%)
SRT treatment session	159 (29%)
Total CBCT	863
Inter-fraction CBCT	556 (64%)
Intrafraction CBCT	307 (36%)
Sex	
Female	21 (30%)
Male	48 (70%)
Median age	66 years (range 27–89)
Diagnosis	
Brain Metastases	32 (46%)
Primary brain cancer	17 (25%)
Primary brain cancer recurrence	10 (14%)
Meningioma	7 (10%)
Vascular malformation	3 (5%)
Type of radiation treatment (RT)	
Conventional RT	
Median dose	40 Gy (range 20–60 Gy)
Median fractions	15 (5–30)
Median target volume	178.6 cc (range 98.8–470.3 cc)
Stereotactic RT	
Median dose	27 Gy (range 12–30 Gy)
Median fractions	3 (range 1–5)
Median target volume	6.15 cc (range 0.7–104 cc)
Median coach rotation	4 (range 0–4)
Median monitor units	2121.7 (range 1037.9–6151.2)
Median overall treatment time	2 min (range 1–5 min)

**Table 2:** Inter-fraction main summary statistical indices.

Population	69 Patients—556 Treatment Sessions—556 CBCT					
	Mean	SD	25%	50%	75%	95% CI
**Longitudinally (mm)**	0.9	0.8	0.3	0.7	1.2	0.82–0.95
**Laterally (mm)**	0.8	0.6	0.4	0.7	1.1	0.82–0.95
**Vertically (mm)**	1.1	0.9	0.5	1.0	1.6	1.05–1.20
**Roll (°)**	0.58	0.49	0.20	0.45	0.9	0.541–0.623
**Pitch (°)**	0.59	0.49	0.20	0.40	0.80	0.545–0.628
**Yaw (°)**	0.67	0.53	0.20	0.50	1.00	0.630–0.718

**Table 3:** Intrafraction main summary statistical indices.

Population	45 Patients—159 Treatment Sessions—307 CBCT					
	Mean	SD	25%	50%	75%	95% CI
**Longitudinally (mm)**	0.3	0.3	0.1	0.2	0.4	0.24–0.34
**Laterally (mm)**	0.3	0.3	0.1	0.2	0.4	0.27–0.35
**Vertically (mm)**	0.4	0.3	0.1	0.3	0.6	0.34–0.45
**Roll (°)**	0.22	0.26	0.10	0.10	0.30	0.182–0.265
**Pitch (°)**	0.33	0.29	0.10	0.30	0.40	0.283–0.376
**Yaw (°)**	0.24	0.26	0.10	0.20	0.30	0.197–0.281

**Table 4:** Intrafraction SGRT main summary statistical indices.

Population		45 Patients—159 Treatment Sessions—307 CBCT					
		Mean	SD	25%	50%	75%	95% CI
**Longitudinally (mm)**	i	0.09	0.18	0.00	0.00	0.10	0.07–0.12
	m	0.45	0.35	0.10	0.40	0.80	0.39–0.50
	f	0.31	0.26	0.10	0.20	0.40	0.26–0.35
**Laterally (mm)**	i	0.07	0.16	0.00	0.00	0.10	0.04–0.09
	m	0.36	0.33	0.10	0.20	0.50	0.31–0.41
	f	0.20	0.23	0.10	0.10	0.20	0.17–0.24
**Vertically (mm)**	i	0.06	0.09	0.00	0.00	0.10	0.04–0.07
	m	0.31	0.31	0.10	0.20	0.40	0.26–0.35
	f	0.22	0.20	0.10	0.20	0.30	0.19–0.25
**Roll (°)**	i	0.025	0.091	0.000	0.000	0.000	0.011–0.039
	m	0.208	0.228	0.000	0.100	0.300	0.173–0.244
	f	0.118	0.144	0.000	0.100	0.200	0.095–0.140
**Pitch (°)**	i	0.036	0.062	0.000	0.000	0.100	0.026–0.045
	m	0.307	0.308	0.100	0.200	0.500	0.259–0.355
	f	0.194	0.208	0.100	0.100	0.300	0.162–0.227
**Yaw (°)**	i	0.039	0.097	0.000	0.000	0.100	0.024–0.054
	m	0.274	0.263	0.100	0.200	0.400	0.233–0.315
	f	0.189	0.229	0.100	0.100	0.200	0.153–0.224

## Data Availability

Research data are not available at this time.
